# EXERTION: a pilot trial on the effect of aerobic, smartwatch-controlled exercise on stroke recovery: effects on motor function, structural repair, cognition, mental well-being, and the immune system

**DOI:** 10.1186/s42466-023-00244-w

**Published:** 2023-05-11

**Authors:** Frederike A. Straeten, Stephanie van Zyl, Bastian Maus, Jochen Bauer, Heiner Raum, Catharina C. Gross, Sabine Bruchmann, Nils C. Landmeyer, Cornelius Faber, Jens Minnerup, Antje Schmidt-Pogoda

**Affiliations:** 1 Department of Neurology, University Hospital Münster, University of Münster, Albert-Schweitzer-Campus 1, Building A1, 48147 Münster, Germany; 2 Translational Research Imaging Center, University of Münster, Münster, Germany; 3 Clinic of Radiology, University Hospital Münster, University of Münster, Münster, Germany

**Keywords:** Stroke rehabilitation, Aerobic exercise, Motor function, MRI, Depression, Fatigue, Immune function

## Abstract

**Introduction:**

Motor impairments are the objectively most striking sequelae after stroke, but non-motor consequences represent a high burden for stroke survivors as well. Depression is reported in one third of patients, the fatigue prevalence ranges from 23 to 75% due to heterogenous definitions and assessments. Cognitive impairment is found in one third of stroke patients 3–12 months after stroke and the risk for dementia is doubled by the event.

Aerobic exercise has been shown to reduce depressive symptoms, counteract fatigue, and improve cognitive functions in non-stroke patients. Furthermore, exercise is known to strengthen the immune system. It is unknown, though, if aerobic exercise can counteract poststroke depression, fatigue, poststroke dementia and poststroke immunosuppression. Therefore, we aim to analyse the effect of aerobic exercise on functional recovery, cognition, emotional well-being, and the immune system. Reorganization of topological networks of the brain shall be visualized by diffusion MRI fibre tracking.

**Methods:**

Adults with mild to moderate stroke impairment (initial NIHSS or NIHSS determined at the moment of maximal deterioration 1–18) are recruited within two weeks of stroke onset. Study participants must be able to walk independently without risk of falling. All patients are equipped with wearable devices (smartwatches) measuring the heart rate and daily step count. The optimal heart rate zone is determined by lactate ergometry at baseline. Patients are randomized to the control or the intervention group, the latter performing a heart rate-controlled walking training on own initiative 5 times a week for 45 min. All patients receive medical care and stroke rehabilitation to the usual standard of care. The following assessments are conducted at baseline and after 90 days: Fugl Meyer-assessment for the upper and lower extremity, 6 min-walk test, neuropsychological assessment (cognition: MoCA, SDMT; fatigue and depression: FSMC, HADS-D, participation: WHODAS 2.0 12-items), blood testing (i.e. immune profiling to obtain insights into phenotype and functional features of distinct immune-cell subsets) and cranial magnetic resonance imaging (MRI) with grid-sampled diffusion weighted imaging, white matter fibre tracking and MR spectroscopy.

**Perspective:**

This study investigates the effect of smartwatch-controlled aerobic exercise on functional recovery, cognition, emotional well-being, the immune system, and neuronal network reorganization in stroke patients.

*Trial registration*

ClinicalTrials.gov NCT Number: NCT05690165. First posted19 January 2023. Retrospectively registered, https://clinicaltrials.gov/ct2/show/NCT05690165

## Introduction

### Rehabilitation from acute stroke

The number of stroke survivors is increasing due to the progress in acute treatment. Despite that, stroke incidence shows a shift to younger age groups [9, 10]. Given the high functional demand in this age group and the remaining lifetime compared to patients, who suffer from a stroke at an advanced age, even subclinical sequelae may have a high impact on quality of life. Moreover, non-motor consequences like poststroke depression or poststroke fatigue represent a burden on national economy. It is reported that 70% of stroke survivors remain limited in their work ability [4].

For stroke rehabilitation there are two relevant forms of neuroplasticity: One that is induced by the lesion itself and one which is induced by physical training [6]. For the latter, the earlier the start of rehabilitation the better the functional improvement [19]. In Germany patients usually start—in- or outpatient—rehabilitation around two weeks after the event and it lasts up to four weeks. Especially the patients with non-obvious deficits tend to fall of the grid afterwards. The transition in care and community rehabilitation especially in the mildly affected patients is not always optimal. The rehabilitation for those patients is often regarded as completed. Despite, they are at a high risk for a second vascular event [19].

### Cognitive impairment after stroke, poststroke-depression, and poststroke-fatigue

Cognitive impairment occurs in more than one third of patients 3–12 months after stroke and concerns impairment in attention, processing speed, executive function, verbal and visual memory, language, and perception abilities [19]. These cognitive deficits can individually persist over years. The risk for dementia is doubled by a stroke [8]. Poor physical activity has been identified as a risk factor for dementia [11]. On the other side, physical activity – even on a low-to-moderate level – exerts a protective effect on cognitive decline [16]. Pathophysiologically, this is potentially explained by a change in cytokines such as enhanced levels of brain-derived neurotropic factor, an increased cerebral blood volume as well as a reduction of depressive symptoms by regular exercise [3].

Depression and anxiety are associated with a poorer functional outcome [19]. The prevalence is difficult to determine but reaches up to one third of stroke survivors at any time after stroke [1]. In studies of adults without a stroke, exercise shows a positive effect on depressive symptoms [12]. Exercise may affect the regulation of elevated cortisol levels which result from the dysregulated hypothalamic–pituitary–adrenal axis in depression [12]. In stroke studies depression often serves as a secondary outcome which is why the effect of exercise is difficult to determine. So far, there are indicators of a positive effect on depressive symptoms but also inconsistent results [19].

Poststroke fatigue may coexist with poststroke depression. It is not consensually defined and characterised by an exhausted feeling, emotional instabilities, increased need for rest and consequently less social participation and a subjective lack of physical or mental energy. The prevalence varies from 35 to 92% due to a wide variability across studies in terms of definition as well as time and method of assessment [2]. No proven therapy exists for poststroke fatigue. From other diseases like multiple sclerosis it is well known that physical exercise improves fatigue symptoms [13].

### Role of the immune system in stroke rehabilitation

The ischemic event in the brain induces a neuroinflammatory process. The excessive activation of the autonomic system causes an immunodepression correlating with a lymphopenia and dysfunctional immune cells. Local immune responses and a local sustained inflammation in the brain, such as formation of neutrophil extracellular traps at the borders of the infarct zone, contribute to further neurological deterioration in the subacute and chronic phase [9, 10]. Anti-inflammatory and neuroprotective treatments in the acute and subacute phase are being investigated, but none of them has proven to be effective yet. In general, the anti-inflammatory effects of regular moderate exercise by affection of the immune regulation are well-known [18]. Regular endurance training changes the phenotype of neutrophils to one which resembles a more anti-inflammatory phenotype. Similar effects are described for monocytes, which differentiate more into the non-classical, less inflammatory phenotype upon regular exercise. Regarding the adaptive immunity, it is known that exercise improves regulatory T-cell-function and –numbers [18].

Taking into account the correlation and assumed contribution of a chronic low-grade inflammatory state to typical lifestyle-associated and cardiovascular diseases and the potential of physical activity to modulate the inflammatory state [18], physical activity might be a link to complement direct treatment of typical stroke consequences.

### Effects of exercise in stroke patients

According to current knowledge, cardiorespiratory training exerts a positive impact on motor and cardiovascular function. Positive effects on the activities of daily living, walking distance and walking speed are reported and the American Heart Association (AHA) recommends endurance training with a duration of at least 20 min on 3–5 days per week [15]. Especially aerobic treadmill or walking training is recommended [19].

After a stroke, patients often enter a vicious circle: Stroke patients reach a deconditioned state which leads to physical inactivity, reduced socialization, and an increased risk of future vascular events (including stroke) [19]. At the same time, the activity level after stroke represents an independent predictor of life satisfaction – including patients who are considered to suffer from a mild stroke [5]. In contrast, stroke rehabilitation programs and routine physiotherapy sessions fail to induce a cardiorespiratory training effect of sufficient intensity and duration [7]. Research indicates that exercise exerts a positive impact on fatigue, depression, mental well-being as reflected by social participation, and health-related quality of life [19]. Whereas very early initiation of exercise (within 24 h after stroke) in the AVERT trial was associated with a reduced chance of a favourable outcome at 3 months, an initiation in the subacute phase (a mean of 11–78 days after stroke) is recommended [19].

As mentioned above, the rehabilitation continuity is not ensured after the in- or outpatient rehabilitation directly after the event. The self-reported and self-perceived amount of daily activity of stroke patients is inflated [14]. The role of wearable devices or apps to provide direct feedback concerning the amount of daily activity has not been elucidated.

## Methods

### Aims of the trial

A deconditioned state in stroke survivors is correlating with motor impairments and interacting with frequent stroke sequela like depression, fatigue, and cognitive impairment. A positive effect of aerobic exercise is generally known, but its implementation in stroke aftercare is challenging [19].

Therefore, the objectives of our study are to determine the effect of regularly walking exercise on the rehabilitation of stroke, especially on motor function, cognition, fatigue, and depression as well as immunological effects. If patients benefit, we aim to further determine estimates about specific subgroups of patients, who benefit most, and the amount of exercise to recommend those patients. In this pilot study, we are also interested in the feasibility of the study concept, patient’s adherence, and organisational requirements for a successful implementation.

### Study description and design

**Fig. 1:**
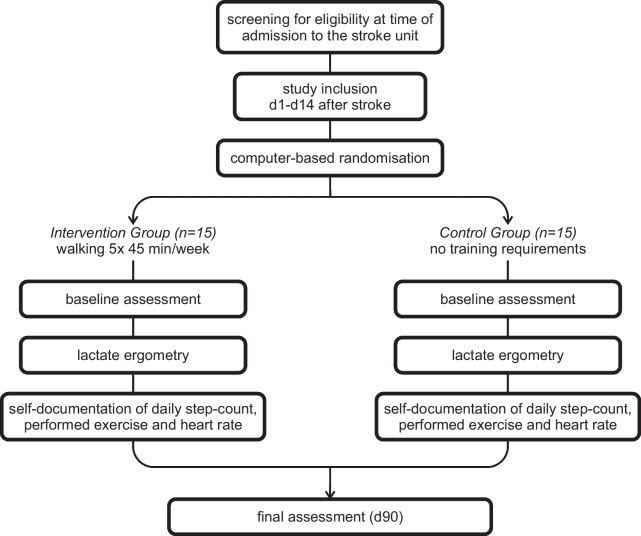
Study flowchart

This is a single-centre pilot study with two groups. Screening for eligibility takes place at admission to the stroke unit and study inclusion in the first 14 days after ischemic stroke. Every participant receives a lactate ergometry with electrocardiogram (ECG) monitoring and is equipped with a wearable device/smartwatch. Participants in the intervention group conduct a heart rate-controlled walking exercise 5 times weekly for 45 min on own initiative for 90 days. Participants in the control group receive no training requirements. All participants document their daily step count as well as performed exercise paper based. The following tests are performed at baseline and at final visit after 90 days: For assessing cognitive functioning the Montreal Cognitive Assessment (MoCA) and the Symbol Digit Modalities Test (SDMT) are administered. Fatigue and depressive symptoms are assessed by the Fatigue Scale for Motor and Cognitive Functions (FSMC) and the Hospital Anxiety and Depression Scale; German Version (HADS-D). To measure the impact of the health condition on a person's ability to participate in daily activities the World Health Organization Disability Assessment Schedule (WHODAS 2.0) is used. Furthermore, blood samples are collected, and MRI scans are conducted. A flowchart of the study is shown below (Fig. 1).

The study is conducted at the University Hospital Muenster, Department for Neurology with Institute of Translational Neurology.

### Arms and interventions

The intervention consists of a regular, heart-rate-controlled walking exercise 5 times per week for 45 min over a period of 90 days after stroke. The heart rate as well as the daily step count is measured by wearable devices/smartwatches. A lactate ergometry is performed for each participant at baseline to determine to optimal heart rate range for exercising. The examination is performed on a bicycle ergometer and the lactate values are assessed by a hand-held meter (Lactate Scout 4). Baseline lactate values are obtained, and patient conduct a step wise load test. Beginning at a load stage of 50 W we perform a step wise increase of 25 W and a duration of 3 min each. At the end of every stage, lactate values and an ECG are recorded with special attention to excitation regression changes or rhythm disturbances. Patients assign a subjective stress level to each stage after being offered a quantitative scale ranging about six items from “low load” to “very strenuous”. The test is ended after the patient reports a subjective exertion. The evaluation is performed by creating a threshold curve and reading the corresponding heart rate at the level of a lactate value of 3 mmol/l. Patients are told to exercise most of the time in a range of 75–95% of this heart rate value. Patients receiving heart-rate-controlling medications (beta-blockers) are told to rather control the load more according to a subjectively light feeling of exertion. This is done for those patients as well whose evaluation failed due to high baseline lactate values. The daily step count and the performed exercise is documented paper based by the participants.

### Eligibility criteria

All patients admitted to the hospital with an acute stroke are screened for eligibility. Patients with severe strokes and those who cannot walk independently are not eligible for the study. As it is a pilot study and because we are interested in feasibility of the study concept, the inclusion criteria are set very broad. We also included patients who would likely have difficulty operating the smartwatch.

### Inclusion criteria for the study:

- ischemic stroke
- initial NIHSS or NIHSS determined at the moment of maximal deterioration 1–18
- age >= 18 years
- pre-stroke independence
- sufficient motivation to exercise regularly

### Exclusion criteria:

- transient ischemic attack
- premorbid motor disability, musculoskeletal disease impairing degree of movement
- balance and transfer function which requires assistance
- cardiac disease not allowing to perform aerobic training
- psychiatric disorders
- inability to give informed consent

### Randomization

We used a computer-generated randomization sequence for 30 patients (https://www.randomizer.org/). This sequence was kept by a study assistance. After inclusion, group allocation was disclosed. After that, all researchers and the patients were aware of group allocation throughout the study process.

### Outcome measures

All assessments are performed at baseline and final assessment after 90 days.

Motor function is measured by the Fugl Meyer-assessment (FMA) for the upper extremity (UE) and the lower extremity (LE) at baseline and final assessment. The FMA-UE is measured in a score with a maximum of 66 for motor function, the FMA-LE reaches a sum score of 34 for motor function. In addition, we will perform the 6-min walk test.

An overall cognitive screening was performed using the MoCA, scores range from 0 to 30. Psychomotor speed was assessed via the SDMT (scores 0–100) in the oral test version. The FSMC (scores 20–100), the HADS-D (score 0–52) and the WHODAS 2.0, 12-item-version (scores 0–48) were administered as self-report questionnaires. All neuropsychological measures were collected in a face-to-face manner by trained personnel. Where possible, a parallel version was used for the 3-month follow-up visit.

To characterize immunological changes, we perform flow cytometry analysis of cryo-asservated peripheral blood mononuclear cells and sera. In our analysis, we focus on the major leukocyte subtypes, effector functions such as cytokine production, cytolytic activity, differentiation and activation levels, and expression of regulatory molecules. A major readout will be comparison of changes in the immune cell profiles between the interventional and control group.

### Magnetic resonance imaging and spectroscopy

Magnetic resonance imaging (MRI) and spectroscopy (MRS) are conducted to reveal structural and metabolic changes following motor therapy. Specifically, tissue structural changes pre and post therapy will be compared via diffusion weighted imaging (DWI) and subsequent fibre tracking. ^1^H-NMR spectroscopy, localized in the stroke area, should reveal metabolic changes between the scan dates. An advantage of all MRI and MRS techniques is the multitude of information that can be derived from single scan sessions. The following is but an excerpt of possible analyses building on the given acquisition scheme, enabling further analyses if new scientific questions arise down the line. Proposed examples for further analyses are given below.

DWI is acquired with a grid-sampled multi-band EPI sequence, using 101 grid elements (vectors) and b-values up to 4000 s/mm^2^. This allows for beyond-tensor modelling of diffusion, in this case generalized q-sampling imaging [20]. Stroke areas will be identified from hyperintense areas in the DWI or a separate T2-weighted FLAIR (fluid-attenuated inversion recovery) sequence, commonly reflecting local edema. Diffusivity parameters in this area, such as mean diffusivity and diffusion anisotropy, can reveal direct information on structural integrity pre and post treatment and can give insight into local tissue remodelling. Deterministic fibre tracking will further uncover larger scale structural white matter changes: Specific interest will be given to fibre tracks extending from the stroke area or those linking affected brain areas across the corpus callosum or via cortico-spinal tracts. Reconstructed tracks will be analysed for fibre count, 3D extent and along-track diffusivity parameters. Further potential analyses could include intraindividual white matter remodelling not confined to or directly associated with the stroke area. Given an atlas registration, these would even enable group comparisons and correlational tractography.

**Fig. 2:**
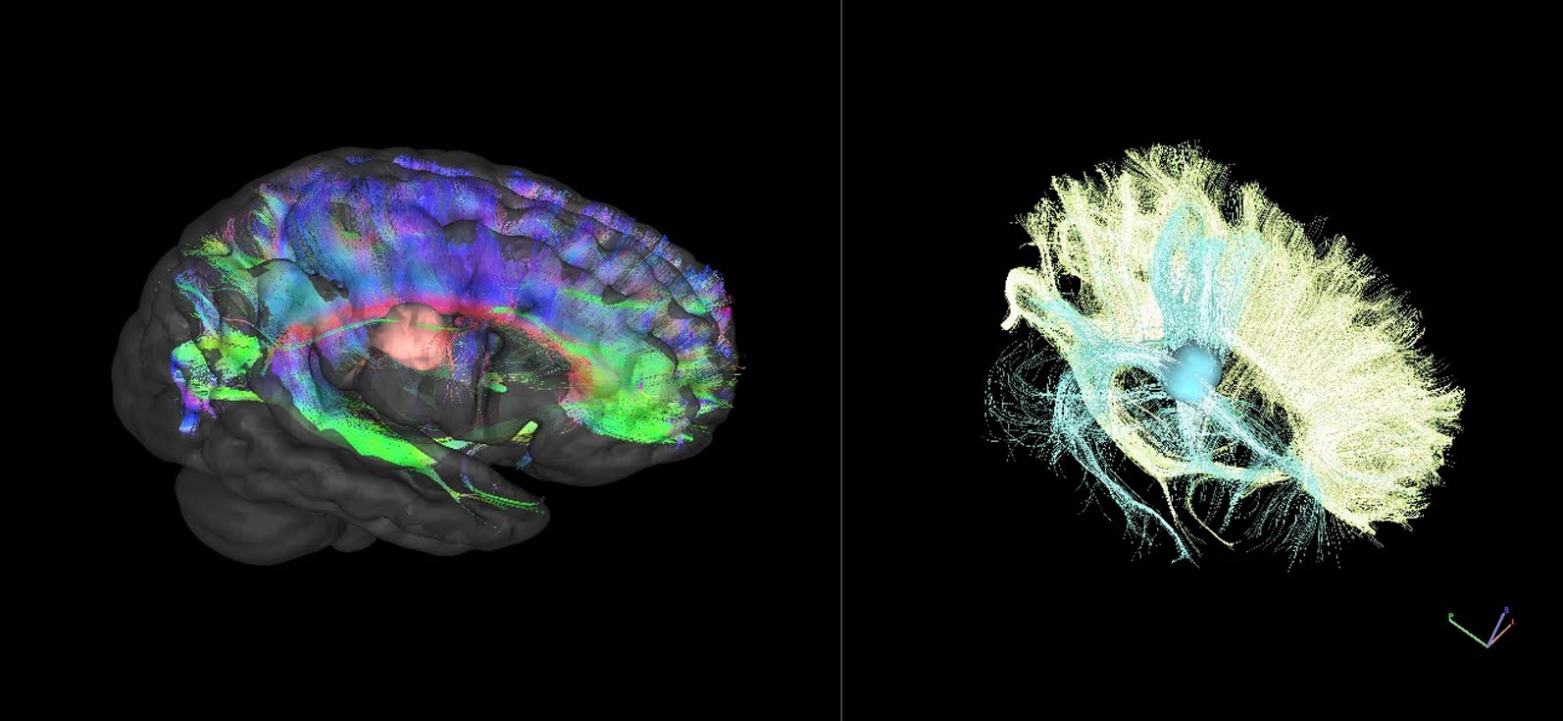
Example images of MRI fibre tracking. The stroke region is highlighted. Left: Colour-coded fibre tracks passing the midline corpus callosum. Right: Corpus callosum fibre tracks are shown in yellow, blue tracks were seeded from the stroke lesion

Localized NMR spectroscopy will reveal the metabolic profile in the stroke area, allowing the study of in situ cellular processes and metabolic conditions. For example, the presence of lactate in the metabolic profile is indicative of anaerobic conditions in the ischemic region, while a decrease in N-acetyl aspartate (NAA) is a sign of acute neuronal damage [17]. Since NMR spectroscopy provides an overview of further metabolites, for example choline-containing compounds, profile analyses may not be confined to lactate and NAA. NMR spectra from the stroke region will be compared to the contralateral healthy area to account for different intra- and interindividual baseline values (Fig. 2).

### Sample size

30 patients will be included in the study. As we perform the first trial to translate our experimental findings into the clinic, no a priori sample size calculation has been performed.

### Statistical analysis

We aim to perform a simple linear regression analysis using the averaged amount of exercise as predictor and measurements on day 90 of each test variable as outcome. Baseline measurements and other possible nuisance variables will be included as additional covariates. As prior analyses, we will analyse the amount of exercise across both groups.

## Perspective

### Strengths

We try to investigate the potential of the use of smartwatches in the rehabilitation of stroke. So far, clinicians recommend regularly exercise—as well as a Mediterranean diet—but have no way to monitor its implementation. After we were allowed to accompany the first patients over 90 days, we were impressed by their dedication. This highlights the underestimated need and motivation of stroke patients to improve their lifestyle.

### Weaknesses

The study requires a high motivation by the participants. Adherence to physical activity without direct reward represents a major challenge. One risk of our study is to include preferentially patients with a high premorbid activity level. We tried to minimize this risk by broad inclusion criteria and setting an individual heart rate zone target. Nevertheless, we cannot adjust for the level of activity patients had before the stroke. Another problem is the operation of the wearable device/smartwatch. Especially older patients face trouble with it and were not able to document their daily activity. Another common challenge for investigating the effects in stroke patients suffering from mild to moderate symptoms is that it is difficult to show the effect. Our sample size is not sufficient to meet this challenge, but we aim to show feasibility of the design to translate the project into larger cohorts.

### Trial Status

Recruitment started in October 2022 and is expected to be complete in the beginning of 2024.

## Data Availability

All data shall be published after termination of the study.

## Abbreviations

AHA: American Heart Association
DTI: Diffusion tensor imaging
ECG: Electrocardiogram
EPI: Echo planar imaging
FLAIR: Fluid attenuated inversion recovery
FMA: Fugl meyer assessment
FSMC: Fatigue Scale for Motor and Cognitive Functions
HADS-D: Hospital Anxiety and Depression Scale; German Version
LE: Lower extremity
MoCA: Montreal Cognitive Assessment
MRI: Magnetic resonance imaging
MRS: Magnetic resonance spectroscopy
mRS: Modified Rankin Score
NAA: N-acetyl aspartate
NIHSS: National Institutes of Health Stroke Scale
NMR: Nuclear magnetic resonance
PSD: Post stroke-depression
PSF: Post stroke-fatigue
SDMT: Symbol Digites Modality Test
TIA: Transient ischemic attack
UE: Upper extremity
WHODAS: World Health Organization Disability Assessment Schedule
